# Real-Time Deduction of Mechanisms and Kinetics Underlying Photocatalytic Water Disinfection: Cell Motility and Particle Tracking

**DOI:** 10.1021/acsestwater.3c00180

**Published:** 2023-08-15

**Authors:** Niraj Ashutosh Vidwans, Kathy Y. Rhee, Pushkar P. Lele, Sreeram Vaddiraju

**Affiliations:** † Artie McFerrin Department of Chemical Engineering, Texas A&M University, College Station, Texas 77843, United States; ‡ Department of Materials Science and Engineering, Texas A&M University, College Station, Texas 77843, United States

**Keywords:** photocatalysis, cell motility, Escherichia coli, cell viability, water disinfection

## Abstract

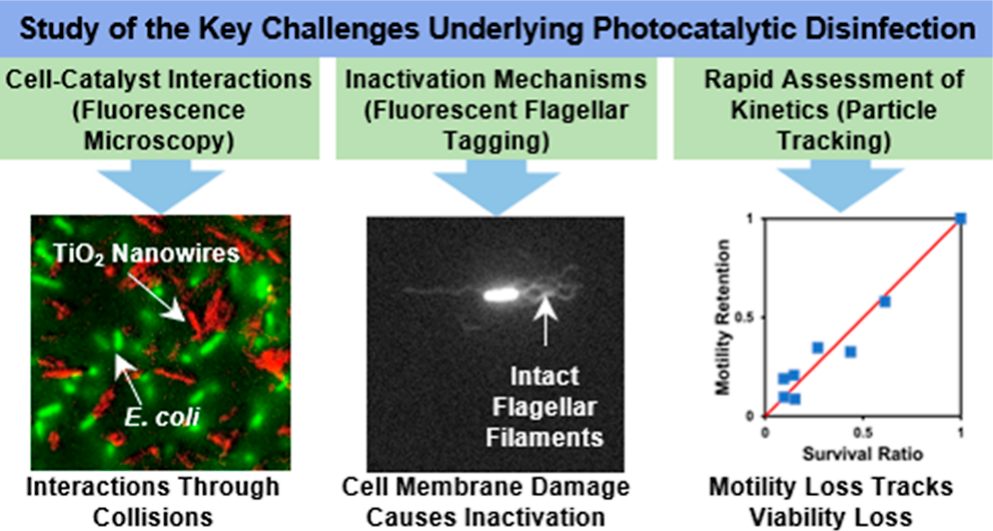

The current methods used to study photocatalysis-assisted water disinfection at a laboratory scale may not lead to process scale-up for large-scale implementation. These methods do not capture the process complexity and address all the factors underlying disinfection kinetics, including the physical characteristics (e.g., shape and size) of the photocatalyst, the light intensity, the form of the catalyst (e.g., free-floating and immobilized), and the photocatalyst–microorganism interaction mode (e.g., collision mode and constant contact mode). This drawback can be overcome using in situ methods to track the interaction between the photocatalysts and the microorganisms (e.g., *Escherichia coli*) and thereby engineering the resulting disinfection kinetics. Contextually, this study employed microscopy and particle-tracking algorithms to quantify in situ cell motility of *E. coli* undergoing titanium dioxide (TiO_2_) nanowire-assisted photocatalysis, which was observed to correlate with cell viability closely. This experimentation also informed that the *E. coli* bacterium interacted with the photocatalysts through collisions (without sustained contact), which allowed for phenomenological modeling of the observed first-order kinetics of *E. coli* inactivation. Addition of fluorescent-tagging assays to microscopy revealed that cell membrane integrity loss is the primary mode of bacterial inactivation. This methodology is independent of the microorganism or the photocatalyst type and hence is expected to be beneficial for engineering disinfection kinetics.

## Abstract 2

Use of cell motility studies and particle-tracking algorithms for evaluating photocatalysis-assisted water disinfection kinetics in real time is presented. Photocatalyst−microorganism interactions, phenomenological models of disinfection kinetics, and disinfection mechanisms are also discussed.

## 1 Introduction

The existing water treatment infrastructure is strained not only due to constantly increasing water demands but also stresses resulting from emerging contaminants such as drug-resistant bacteria, pharmaceuticals, and per/poly-fluoroalkyl substances.^1^ Advanced oxidation processes (AOPs), such as heterogeneous and homogeneous photocatalysis, have the potential to complement the current methods employed for treating water in the removal of these emerging contaminants and alleviate some of the water stresses.^2,3^ Heterogeneous photocatalysis typically involves the ultraviolet (UV) and/or visible-light excitation of solid semiconductor photocatalysts added to the contaminated water that needs to be purified.^4,5^ The excitation of the photocatalysts leads to the creation of reactive oxygen species (ROS) that oxidize the contaminants.^6^ The ROS generated during heterogeneous photocatalysis include hydroxyl radicals (OH), superoxide radical ions (O_2_^^•^–^), and hydrogen peroxide (H_2_O_2_).^4,7−9^ The supply of photocatalysts for removing contaminants from water during heterogeneous photocatalysis is accomplished in many ways, including deployment in the form of slurries or suspensions, or by immobilizing them on reactor walls or other neutral catalyst supports.^10,11^ The primary advantage of heterogeneous photocatalysis over homogeneous photocatalysis is the potential to recover, regenerate, and reuse the photocatalyst.^12^

The literature on heterogeneous photocatalysis primarily focuses on the development and optimization of the photocatalyst material chemistry, with only a minor focus on photocatalysis process development. In particular, a wide variety of heterogeneous photocatalysts has been employed for disinfecting water and inactivating bacteria, fungi, and viral pathogens present in water.^8,13,14^ Numerous studies have also elucidated the factors controlling the kinetics of photocatalytic disinfection in bacterial cultures, both at laboratory scales (comprising of systems of tens of milliliters)^15−17^ and at pilot-scale deployments (of up to 10 L).^10,18^ A noteworthy conclusion from these studies is that the disinfection kinetics during heterogeneous photocatalysis are relatively slow when compared to homogeneous AOPs, with disinfection requiring times on the order of a few hours or more.^12,19,20^

Further engineering of the kinetics of photocatalytic disinfection may not be possible with the current experimental methodology as most of the laboratory-scale studies are narrow in focus and do not completely capture the complexity of the photocatalysis process. In addition to the size, shape, and chemistry of the photocatalyst, the method of deployment of the photocatalyst and the mode of interaction between the photocatalysts and the microorganism play a major role in the determination of the disinfection kinetics. For example, TiO_2_ nanoparticles suspended in water (e.g., Aeroxide P25, diameter: ∼25 nm^21^) likely inactivate bacterial cells faster compared to TiO_2_ nanowire suspensions (e.g., anatase nanowires, diameters: ∼100 nm and lengths: ∼2–10 μm, Afreen et al.^22^) owing to the difference in their sizes and shapes.^12,22^ It is generally postulated that multiple TiO_2_ nanoparticles adsorb on the microorganism (e.g., *Escherichia coli*) surfaces.^5,23,24^ This constant contact mode of interaction between the photocatalyst and the microorganisms may lead to disinfection kinetics that are relatively faster than those observed with TiO_2_ nanowire photocatalysts. This is unlike the case of TiO_2_ nanowires, whose lengths may be similar to (or larger than) the dimensions of the microorganisms.^22^ Similarity in dimensions between the nanowire catalysts and microorganisms may prevent the direct adsorption of the nanowire on the microorganism surface or vice versa.^24^ Therefore, collisions may be the mode of interaction between the photocatalyst nanowires and the microorganisms as previously described by Dalrymple et al. and van Grieken et al.^24,25^ However, experimental tests of the underlying assumptions in these models are not always available. This may either limit the applicability of the models developed or render them inaccurate. For example, kinetic models developed using nanoparticles as photocatalysts may not be accurate when nanowires are employed as photocatalysts as both the dimensions and the shapes of the photocatalysts are different in both cases.^24,26−29^

The mode of interaction between the photocatalyst and the microorganisms also has a major impact on photocatalysis process development. Amounts of the photocatalyst recovered may be different depending upon the sizes and shapes of the photocatalysts. For example, in a previous work, our group observed that gravity-assisted settling and centrifugation lead to the recovery of 77% of Aeroxide P25 (TiO_2_) nanoparticles and only 57% of TiO_2_ nanowire photocatalysts.^12^ Moreover, the constant contact mode of interaction may foul the photocatalysts faster than the collision mode of interaction and complicate the photocatalysis process development further. Altering the mode of supply of the catalyst from nanoparticle/nanowire suspensions in water to photocatalysts supported on neutral substrates only adds to the complexity associated with the photocatalysis process development described above.

In short, a key challenge to both experimentally validating the assumptions underlying the different models describing the kinetics of disinfection and engineering the disinfection kinetics is the lack of approaches to measure the microorganism inactivation in situ rapidly and the mode of photocatalyst–microorganism interactions during photocatalysis. Current approaches to measure the rate of disinfection primarily rely on drawing small samples from photocatalytic reactors at different time points and culturing the cells on soft-agar nutrient media over several hours.^5,30^ The rate of decrease in the number of colony-forming units (CFU) upon photocatalysis indicates the kinetics of disinfection. As this approach needs extended incubation times, it fails to account for cellular adaptation on the culture media that might occur once the stressor (i.e., light exposure in the presence of a photocatalyst) is removed. This can result in underprediction of the disinfection rates. In some cases, the CFU approach may overpredict the disinfection rate, as the shearing forces involved in plating can eliminate otherwise undamaged cells merely because of the adsorbed nanoparticles.^30^ Flow cytometry partly alleviates some of these concerns, but it does not provide in situ or real-time information about individual microorganism cell physiology and the cell–photocatalyst interactions.^30^ Ex situ electron microscopy can reveal outer membrane damage in Gram-negative bacterial species if it is substantial; however, it lacks the ability to reveal inner membrane damage, which is usually the main cause of photocatalytic cell inactivation.^26,31−33^

A key aspect of cellular membrane damage is the loss of the electrochemical potential that powers cellular activity.^23,24,26^ Known as the proton-motive force (PMF), this energy source powers adenosine triphosphate (ATP) synthesis, efflux activity, and in particular, motility. Bacteria, such as *E. coli*, swim with the help of flagella.^34−36^ Each flagellum consists of a helical extracellular filament that is several microns long and ∼20 nm in diameter.^37,38^ The filament is rotated at its base by a transmembrane protein motor powered by the PMF.^39−42^ Disruption of PMF inhibits motility immediately, and hence motility has been popularly employed to quantify changes in the PMF under different stressors.^43^ Therefore, addressing whether motility loss strongly correlates with cell viability loss will allow for the real-time quantification kinetics underlying photocatalysis-assisted disinfection.

In this context, the aim of this work is to employ optical (phase) microscopy, particle-tracking algorithms, fluorescent-tagging assays, and fluorescence microscopy for a threefold purpose: (i) observe and quantify in real-time the motility loss of bacteria undergoing photocatalysis and deduce whether motility loss rates correlate with viability loss rates, (ii) observe in real-time the mode of interaction of *E. coli* with the nanowires and use the obtained data to deduce a phenomenological model explaining bacterial inactivation kinetics, and (iii) deduce a mechanism underlying inactivation of bacteria undergoing photocatalysis. Here, the strong correlation observed between motility loss rates and viability loss rates is expected to lead to the use of the former as a marker for quantifying in real-time kinetics of disinfection by photocatalysis. For this study, *E. coli* served as the model bacteria, with TiO_2_ nanowires (produced using the solvo-plasma method^12,22,44,45^) serving as the photocatalyst and ultraviolet-A (UV-A) lamps providing for the photoactivation of the nanowires. The micron-scale lengths of the TiO_2_ nanowires employed as photocatalysts allowed for observing both *E. coli* and nanowires by optical microscopy.

## 2 Materials and Methods

### 2.1 Materials and *E. coli* Cultures

Porous TiO_2_ nanowires employed as photocatalysts in this study were obtained from Advanced Energy Materials, LLC (Louisville, KY, USA). These TiO_2_ nanowires were synthesized using a solvo-plasma approach with atmospheric pressure plasma jets. The synthesis method for the nanowires has been reported elsewhere.^44−46^ For the photocatalysis experiments, the UV-A light source employed was a SunLite 20 W, 15 Lumens Blacklight (SunLite, Brooklyn, NY, USA). The light source and the *E. coli*-containing water samples were enclosed within a cardboard box lined with aluminum foil for performing the photocatalysis experimentation. Lennox broth agar plates (CulGenex Lennox Broth (LB) Agar Plated Media, Hardy Diagnostics, Santa Maria, CA) were used for spread-plating of cells. Purified deionized (DI) water from the PURELAB Chorus 1 Water Purification System, (ELGA LabWater) was used as the reaction medium. The water was autoclaved for 20 min at 121 °C prior to its use as the reaction medium. A motility buffer (MB) solution was prepared from the above-mentioned purified DI water. The MB consisted of 10 mM potassium phosphate, 67 mM NaCl, 0.1 mM EDTA, 1 μM methionine, and 10 mM sodium lactate, pH ∼6.95.^47^ Minor modifications to the Bioptechs Delta-T heated culture dishes were made to use them as both the photocatalytic reactors and experimental systems for quantifying the motilities of *E. coli*. These were prepared using the following procedure. A 600 μL aliquot of TiO_2_ nanowire suspension containing 0.1 g/L of TiO_2_ nanowires in DI water was introduced into each of the Bioptechs Delta-T heated culture dishes, followed by air drying. This led to the formation of a uniform coating of the nanowires on the top surface of the Delta-T dish (henceforth referred to as “photocatalyst-coated Delta-T dish” in this article).

The AW405 wildtype *E. coli* strain, which is a derivative of *E. coli* K-12, was employed for all motility and growth assays. A derivative of AW405, HCB1737, was employed to fluorescently label and visualize the flagella. The latter strain carries a cysteine residue in the *fliC* allele,^48,49^ which helped label the flagellin proteins with a maleimide-based dye. This helped determine the presence/absence and integrity of flagella in *E. coli* upon photocatalysis. However, for complete cell visualization during fluorescence microscopy observations of cell–catalyst interactions, HCB1737 transformed with a *ptrc99A-eYFP* plasmid was employed. This allowed for clearly distinguishing *E. coli* from the nanowires during fluorescence microscopy studies.

Culturing of *E. coli* was performed using the following protocol. First, fresh colonies were streaked from glycerol stocks on LB agar plates and incubated overnight. Individual colonies from the streaked plates were used to inoculate overnight cultures. These overnight cultures were grown in 5 mL of Tryptone Broth (TB) at 30 °C in a rotary shaker. After 15–18 h, fresh day cultures were started by diluting the overnight cultures at a 1:50 ratio in 10 mL of TB. To induce the expression of enhanced yellow fluorescent protein (eYFP), 100 μg/mL ampicillin and 100 μM isopropyl β-d-1-thiogalactopyranoside (IPTG) were added for the observation of cell–catalyst interactions using fluorescence microscopy. The day cultures were grown at 33 °C to an optical density (OD_600_) of 0.6 in a rotary shaker set at 170–200 rpm. A 1 mL aliquot from the day culture was then centrifuged at 1000 residual centrifugation force for 7 min. The supernatant was replaced with purified DI water and the cell pellet was resuspended gently with a micropipette tip. The cells were pelleted via centrifugation and washed in water in this manner twice. DI water was preferred over MB for the experiments as the latter contains ions such as nitrates and phosphates. These ions may counteract the photocatalytic effect of TiO_2_ by reacting with ROS.^50^ The final cell pellet was resuspended in 1 mL of purified DI water to obtain a cell concentration of ∼10^8^ CFU/mL. This resuspension was further diluted 5-fold in purified DI water to obtain a working cell suspension. The protocol followed for the culturing of cells, along with the protocol for the simultaneous observation of cell motility and cell viability loss, is summarized in Figure S1 of the Supporting Information. The cell motility loss and cell viability loss quantification protocols are discussed in the following sections.

### 2.2 Real-Time Study of Cell Motility and Viability under Photocatalytic Action (Phase Microscopy Studies)

For performing optical microscopy studies for observing *E. coli* motility during photocatalysis, 300 μL of the working cell resuspension was transferred into a photocatalyst-coated Delta-T dish. The dish was then covered with a 22 mm circular coverslip to decrease evaporation and hydrodynamic flows. Finally, the dish was affixed on a glass slide with double-sided tape (Figure S2, Supporting Information) and mounted on a Nikon Optiphot 2 microscope stage. Cells were observed using a 10× phase objective and motility was recorded with a charge-coupled device (CCD) camera (IDS imaging, UI-3240LE). The culture dish was maintained at room temperature for the duration of the experiment. The suspension was exposed to UV-A light and motility was recorded at various pre-determined time points to study the change in motility over the course of photocatalysis. Simultaneously, small volumes of the cell suspension were also withdrawn from the dish at various time points and introduced into tunnel slides, which were prepared by sticking a glass coverslip (22 × 22 mm, 1.5 no. Fischer Scientific) to a glass slide with the aid of two spacers made of 3M Scotch permanent double-sided tape.^49^ The tunnel slides enabled us to repeat the motility measurements in the absence of the nanowires, which helped minimize particle-tracking errors as discussed in the next section. Control measurements were performed in a similar manner in the absence of either UV illumination (termed “dark control”) or the photocatalyst coating (termed “clear control”) or both (termed “negative control”). Each treatment involved at least three biological replicates.

In conjunction with these motility experiments, the changes in cell viability during photocatalysis were also determined by withdrawing small samples of cell suspensions undergoing photocatalysis at various pre-determined time points and plating them on the LB agar plates for overnight incubation at 37 °C. Control measurements were also performed in a similar manner. For each plating, a few microliters of cell suspensions from the Delta-T dish were drawn and serially diluted up to a 1:10,000 dilution in MB and multiple dilutions were spread-plated. The colonies grown on these plates were counted manually after incubation overnight.

### 2.3 Quantification of Cell Motility Using Particle-Tracking Algorithms

Well-established particle-tracking algorithms^51,52^ were employed to quantify changes in cell motility during the course of photocatalysis. Briefly, the location of each cell, ***r***_***n*,*i***_, was determined with a brightness-weighted centroid detection approach in each *i*th frame of the video. Here, *n* identifies the cell. Cell locations in subsequent frames were then linked to obtain the trajectory for each individual cell.^52^ For each cell, the instantaneous velocity was calculated as ***v***_***n***_**(τ) = (*r***_***n*,*i*+1**_ – ***r***_***n*,*i***_**)** × **fps**, where fps is the camera frame rate. Mean speed was calculated by averaging the instantaneous speeds over the duration for which each cell was observed. Cells that had an average speed <6 μm/s threshold were excluded from further analysis. This threshold was chosen to exclude cells undergoing only Brownian motion due to hydrodynamic effects. In short, cells that moved at speeds higher than the threshold were labeled as motile. The labeling was also validated using visual observations. Using this approach, tracks of all cells were plotted and their average speeds were also calculated. The motile fraction (MF) was then calculated using eq 1
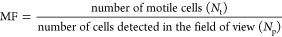
1

The motility retention ratio was also estimated by dividing the post-treatment motile fraction value at a given time point, MF(*t*), by the pre-treatment value, MF_*i*_, according to eq 2
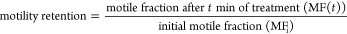
2

### 2.4 Flagellar Labeling Studies Using Fluorescence Microscopy

As mentioned briefly in the Section 2.1 of Materials and Methods, the HCB 1737 strain of *E. coli* was used to determine the effect of photocatalysis on the flagellar filaments. These cells were cultured and washed using the procedure outlined in Section 2.1. The washed cells were suspended in 1 mL of water and subjected to photocatalysis in the Delta-T dish setup described above for a duration of 1 h. Next, the cells were washed repeatedly in MB and centrifuged to obtain a cell pellet. 1 μL of Atto 514 maleimide dye (Sigma-Aldrich) was then added to the pellet and the cells were incubated for ∼30 min in a rotary shaker. The cells were then repeatedly washed in MB, and finally resuspended in 200 μL of MB. The cells were then observed using total internal reflection fluorescence (TIRF) microscopy. A Nikon Ti-E inverted microscope equipped with a LED white light source (SOLA SE Light Engine, Lumencor, Inc.) and a 60× TIRF objective (Nikon, Inc.) was used for these observations.

### 2.5 Observation of Cell–Nanowire Interaction Mode Using Fluorescence Microscopy

To determine how the nanowires interacted with the cells, yellow fluorescent protein (eYFP) expression from an IPTG-inducible vector (*ptrc99A*) was performed on HCB 1737 cells. This enabled the visualization of the cells and nanowires simultaneously under fluorescence illumination in a Delta-T dish coated with the nanowires. It is essential to add here that the TiO_2_ nanowires were observed to luminesce in the 460–475 nm regime upon excitation.^53^ This voided the need to add additional fluorescent tags to the nanowires for their visualization under fluorescence microscopy. As mentioned above, a Nikon Ti-E microscope was used for these observations. However, the nanowires were viewed with cyan excitation and recorded in the green channel (480/40 bandpass filter, AVR optics), while the cells were excited with yellow illumination (514 nm) and visualized in the yellow channel (525–555 nm, Chroma Technology Corp.).

## 3 Results

### 3.1 Effect of Photocatalytic Treatment on Motility and Viability of *E. coli* Cells

**Figure 1:**
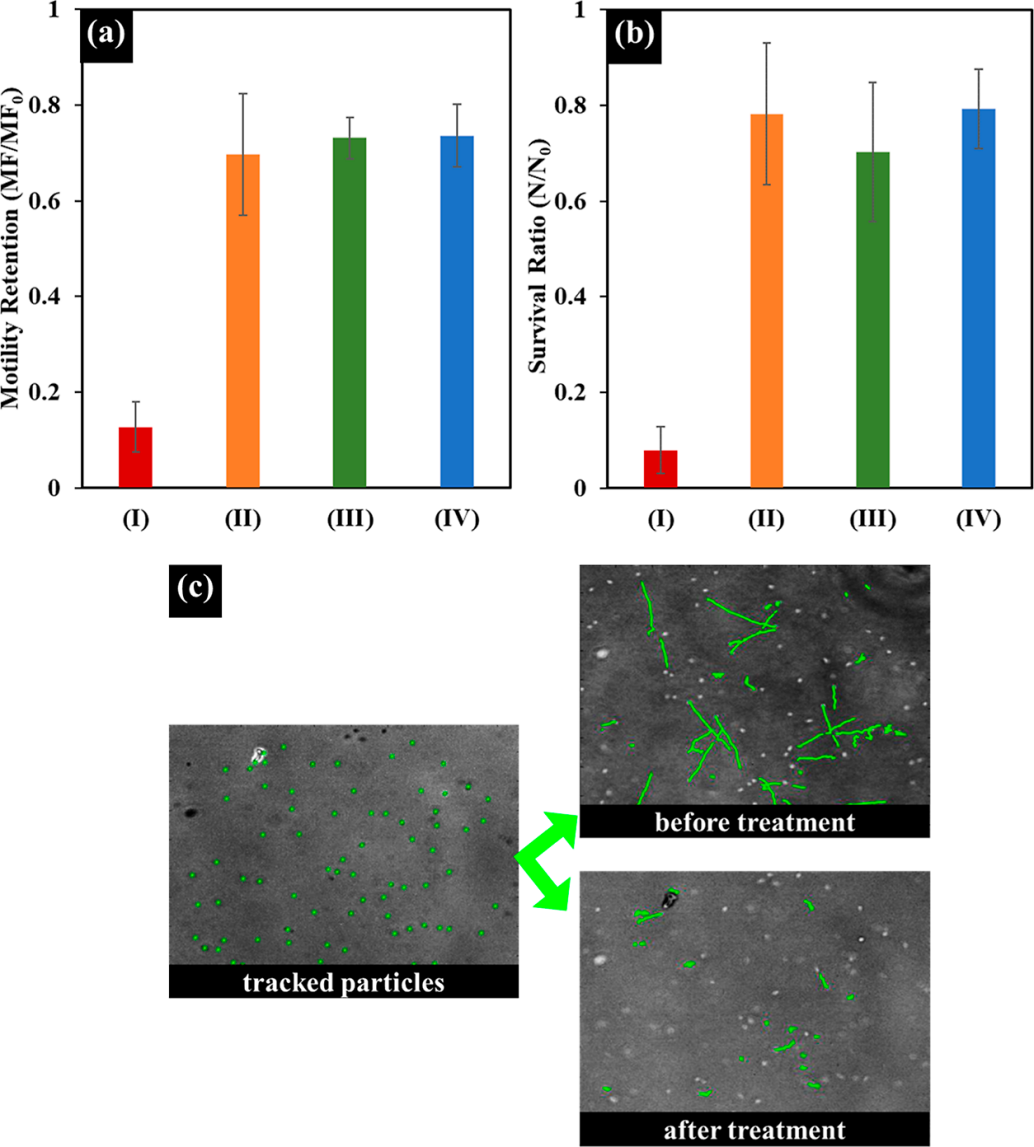
(a) Motility retention and (b) survival ratio of cells undergoing the following treatments for a period of 60 min each: (I) photocatalysis with both UV-A and TiO_2_ nanowires present, (II) clear control with only UV-A exposure, (III) dark control with only exposure to TiO_2_ nanowires, and (IV) negative control with exposure to no UV-A light and no TiO_2_ nanowires. The error bars in (a,b) represent the standard deviations obtained from three biological replicate experiments. (c) Examples of particles identified and tracks generated by the particle-tracking algorithms before and after treatment in the test case with both nanowires and UV-A present.

A primary observation made from these experiments was the loss of cell motility upon photocatalytic treatment of *E. coli* using immobilized nanowires. This can be qualitatively observed in Videos S1 and S2 (Supporting Information) which depict the bacteria and nanowires in situ, before and after 1 h of UV-A exposure. As evident in these videos, a large number of the cells undergo only simple Brownian motion after the treatment. The UV-A/TiO_2_ nanowire treatment caused an average of about 87% of the cells to lose their motility [Figure 1a (bar “I”)]. Here, cells are considered motile only if their speeds exceeded 6 μm/s (see Section 2.3 in Materials and Methods). When the cells were only exposed to UV-A light in the absence of the photocatalyst (clear control), only 30% of the cells lost motility [Figure 1a (bar II)]. In experiments involving exposure of *E. coli* to the photocatalyst nanowires in the absence of UV-A (dark control), about 27% of the cells lost motility [Figure 1a (bar-III)]. Finally, in a negative control, where the UV light and the photocatalyst were both absent, about 26% of the cells lost motility [Figure 1a (bar IV)]. It is believed that the loss of motility in the negative control is due to adverse osmotic effects on the cells caused by DI water, which is reflected in the basal values in all the controls. The motility behavior associated with all the control experiments can be viewed in the Supporting Information; specifically, clear control (Videos S3 and S4), dark control (Videos S5 and S6), and negative control (Videos S7 and S8). In summary, the presence of only DI water, exposure to only UV-A light, and the proximity of *E. coli* to only TiO_2_ nanowire photocatalyst in the absence of any UV-A activation did not differentially impact motility. Significant inhibition in motility was only observed in the UV-A/photocatalyst treatment.

The second observation made was the loss of cell viability upon photocatalytic treatment of *E. coli* using immobilized nanowires. The CFU counts decreased after the photocatalytic treatment of *E. coli* as shown in Figure 1b [bar (I)], with ∼92% of the cells losing viability. In contrast, only ∼22–30% of the cells were inactivated in the control studies [Figure 1b, bar (II)–bar (IV)]. Two-tailed student’s *t*-tests confirmed that the differences between the test case and all three control experiments are statistically significant, with *p*-values < 0.01 for all comparisons in motility and viability of the cells (the differences in means were considered to be statistically significant for *p*-values < 0.05).

### 3.2 Nature of Cell–Photocatalyst Interactions

The similar appearance of nanowires and bacterial cells in phase microscopy made it challenging to determine if the nanowires adsorbed onto the bacterial cells during photocatalysis. Hence, fluorescence microscopy was employed to observe the catalyst–cell interactions. As mentioned above, the cells were observed by expressing and exciting eYFP. The luminescence of the nanowires was imaged in the green emission channel (see Section 2.5 of the Materials and Methods). There was little spectral overlap between the signals from the nanowires and the cells.

**Figure 2:**
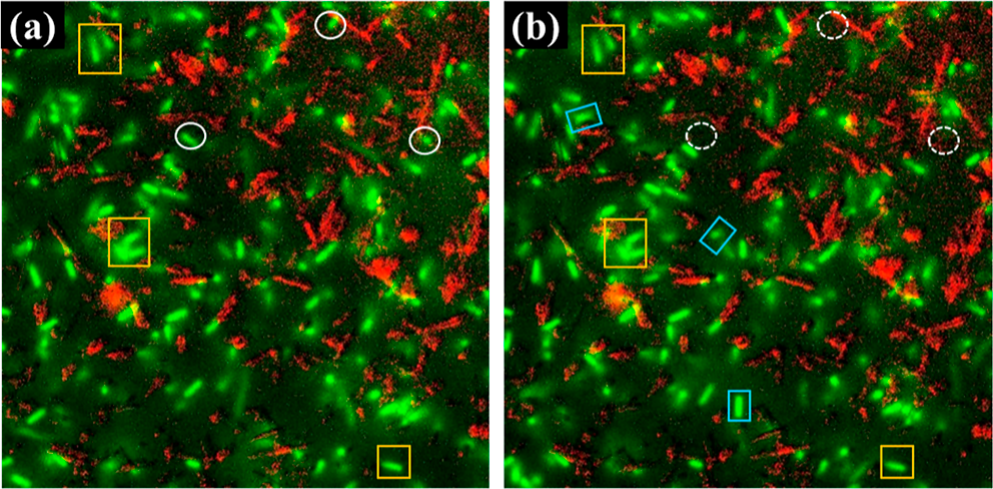
Overlay of nanowires (in red) and bacteria (in green) observed using fluorescence microscopy at (a) beginning of observation and (b) 6 s later. Some cells seen initially can be seen to have moved to another location (shown in white circles) while some new cells can be seen to have appeared in the frame at the end of 6 s (represented by blue rectangles). Yellow squares show examples of the locations of non-motile cells. Also, see Video S9 in the Supporting Information.

Figure 2 shows still images of a video of the same region at two different time points (the complete video is included in Video S9 of the Supporting Information). The images depict a small number of cells immobilized on the glass surface close to the nanowires; however, no direct adsorption of nanowires on the *E. coli* cells was evident (shown in yellow squares in Figure 2). A large fraction of the cells did not appear to adhere to the nanowires at all. As shown in Figure 2, a significant number of cells both moved out of the region of interest and moved into the region of interest. For example, cells shown in white circles in Figure 2a moved out of the region of interest. This is clearly evident from the empty hashed circles in Figure 2b captured 6 s after Figure 2a was captured. These hashed circles represent the original locations of the cells in Figure 2a before they moved out. Similarly, cells that moved into the region of interest are indicated by blue rectangles in Figure 2b. These cells were not visible in the image captured 6 s prior.

### 3.3 Nature of Cell Damage during Photocatalysis

The loss of cell motility observed above can be attributed to the ROS generated during photocatalysis damaging either cell membranes or the proteins within the flagellar filaments. The flagellar filaments are only ∼20 nm in diameter, too thin to be observed by optical microscopy. To test whether flagella were damaged, a derivative of AW405 (HCB 1737) was employed. This strain of *E. coli* carries a cysteine residue in the extracellular flagellar protein, which makes it possible to label the filaments with a maleimide-based fluorescent dye and enables the visualization of flagellar filaments using fluorescence microscopy.^37,48,49,54^

**Figure 3:**
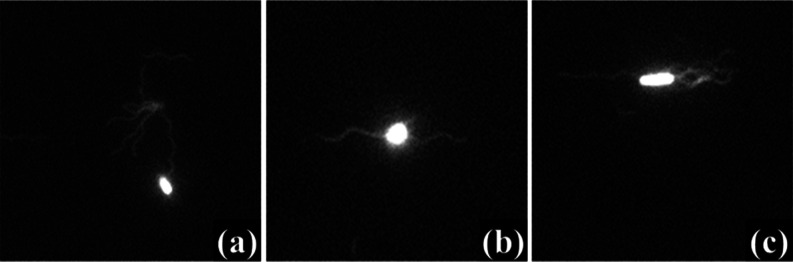
(a–c) Optical micrographs of fluorescent-labeled flagella of treated cells. The flagellar filaments remained intact and predominantly associated with the cell body (bright ellipsoids). Also, see Video S10 in the Supporting Information.

As depicted in Figure 3 (and Video S10 in the Supporting Information), the flagellar filaments remained intact and predominantly remained attached to the cells after 1 h of photocatalytic treatment. The filaments observed in Figure 3 were similar to the flagellar filaments observed in undamaged cells and reported previously.^38^ Therefore, the loss in motility during photocatalysis is attributable to the damage caused by ROS to the cell membrane and the ensuing PMF dissipation.^34−36,43^ This notion is further supported by the close agreement between the quantitative decrease in motility and the viability during photocatalysis (Figure 1). Together, these results suggest that motility loss can be employed to track the loss of cell viability in *E. coli* in real time. As the loss in swimming speeds can be quantitatively obtained for single cells, the assay presented in this work is a powerful approach to deduce the underlying cell inactivation mechanism and cellular adaptation during photocatalysis.

### 3.4 Kinetics of Motility and Viability Losses

**Figure 4:**
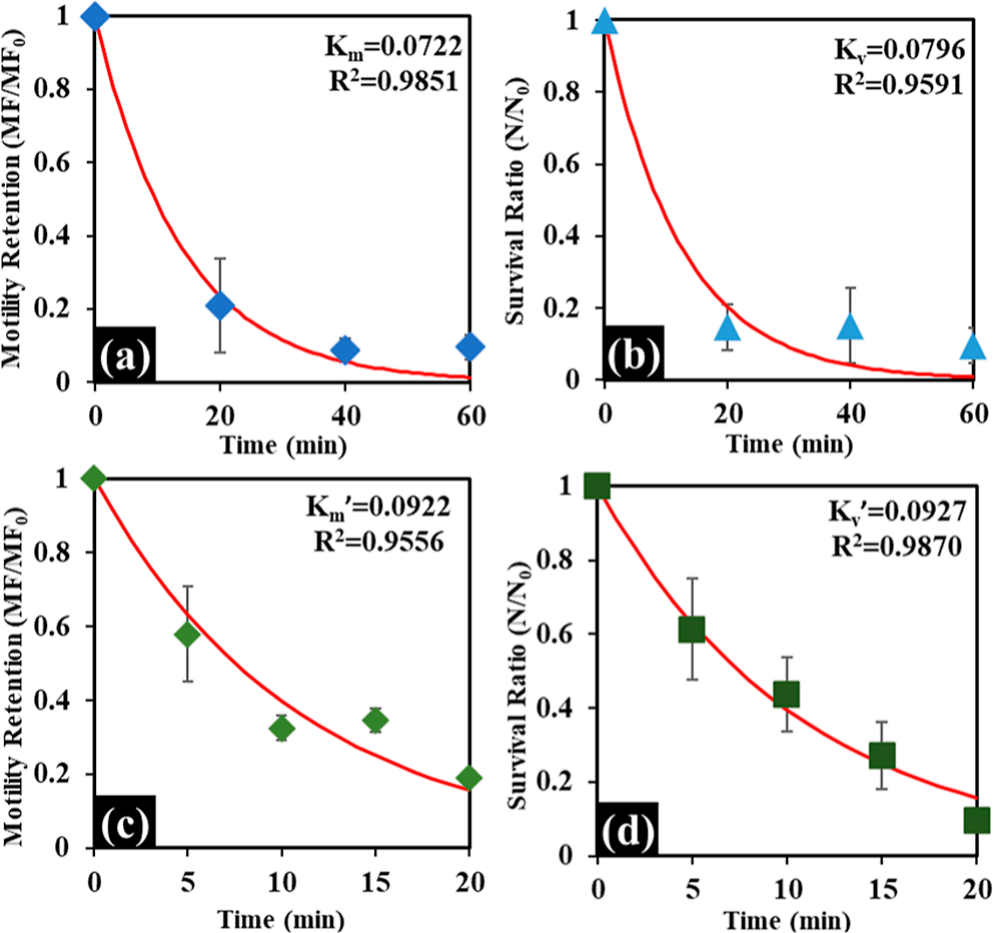
(a,b) Motility retention ratios and survival ratios, respectively, under photocatalytic treatment. Samples were drawn every 20 min. (c,d) Motility retention ratios and survival ratios, respectively, with samples drawn every 5 min. All the kinetic data were fitted to a rate law, which is first order in nature for the survival ratio/motility retention ratio (shown as the red line). Values of the first-order rate constant (*K*_v_/*K*_v_^′^ for viability loss and *K*_m_/*K*_m_^′^ for motility loss) obtained from the best fit of the data are provided in each figure. The experimental data reported are average values of three biological replicate experiments. The error bars represent the standard deviations obtained from these three replicate experiments.

Time-series measurements of motility and viability loss aided in quantifying the kinetics of change in the two physiological parameters during the photocatalytic treatment. Briefly, the experiments described in Section 3.1 were performed at specific time intervals over the duration of 1 h (see Section 2.4 of Materials and Methods). Figure 4a,b indicates motility and viability losses, respectively, upon sampling the cell suspension every 20 min. Both motility and viability decreased exponentially with time. Using mean squared fitting, a good fit of the data with the equation *N*/*N*_0_ = e^–*Kt*^ was observed. Here, *N* is the concentration of motile cells (or viable cells) at time *t* in minutes, *N*_0_ is the initial concentration, and *K* is the decay rate constant observed in these experiments. The fits to the motility and cell viability data yielded similar decay constants: *K* = *K*_m_ = 0.0722 min^–1^ for motility loss and *K* = *K*_V_ = 0.0796 min^–1^ for viability loss. Moreover, the correlation between the motility and viability losses was observed to be independent of the sampling interval. Short-time sampling every 5 min indicated similar kinetics (Figure 4c,d): *K* = *K*_m_^′^ = 0.0922 min^–1^ and *K* = *K*_V_^′^ = 0.0927 min^–1^. The error bars in these figures represent the standard deviations obtained from three biological replicate experiments.

### 3.5 Photocatalytic Disinfection Kinetics in Suspended versus Immobilized Catalysts

**Figure 5:**
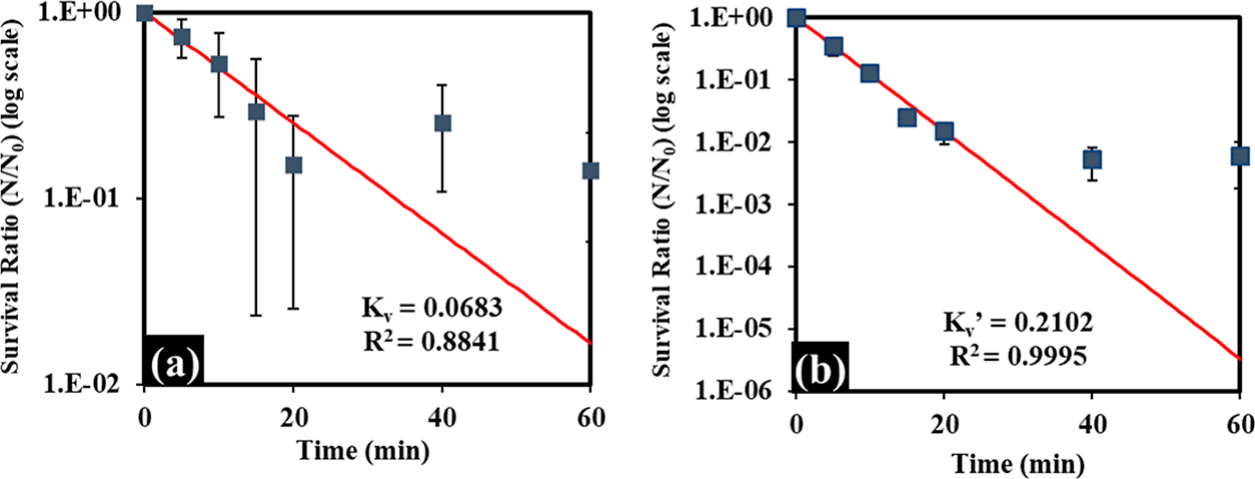
Comparison of the kinetics of cell inactivation with (a) immobilized photocatalysts and (b) suspended photocatalysts. Values of the first-order rate constant (*K*_v_ and *K*_v_^′^) are provided in the plots and indicate faster kinetics in the case of suspended photocatalysts relative to the immobilized photocatalysts. The experimental data reported are average values across three biological replicate experiments. The error bars represent the standard deviation observed in the three replicate experiments.

To ensure that the results presented above (namely, the kinetics of inactivation of *E. coli* and the cell–photocatalyst interaction mode) are applicable for large-scale deployment of photocatalysis, the kinetics of *E. coli* inactivation in 50 mL-sized photocatalytic reactors employing suspensions of photocatalyst nanowires were also obtained. To accomplish this task, the photocatalysis experiments were repeated in 50 mL quartz beakers by exposing suspensions of the TiO_2_ nanowires and the *E. coli* cells to UV-A light under constant magnetic stirring. This procedure was previously reported in detail by our group.^12,22^ The resultant kinetics of *E. coli* inactivation are indicated in Figure 5b (for comparison, the kinetics of *E. coli* inactivation at the T-dish-scale are plotted in Figure 5a). Initially, the viability decayed exponentially with a decay constant (*K*_v_^′^ = 0.2102), but at later times (i.e., time >20 min), the decay rate was slower (Figure 5b). The trend observed is consistent with the results presented in Figure 5a. However, the kinetics of disinfection are significantly higher when photocatalysts are employed in the suspension form (Figure 5b) relative to those observed when the photocatalyst is in an immobilized form (*K*_v_ = 0.0683, Figure 5a).

## 4 Discussion

Overall, the results presented indicate that motility loss closely tracks the loss of cell viability in *E. coli* cultures during photocatalysis (Figures 1 and 4). Hence, motility could be used to monitor changes in viability in real-time, circumventing long wait times and cellular adaptation problems associated with a spread-plate methodology involving prolonged incubation on soft-agar plates. Moreover, the results also indicate that loss of motility is due to changes in intracellular activity during photocatalysis and not due to the extracellular flagellar filament damage (Figure 3). As cell viability appeared to decrease at the same rate as motility (Figure 4), it is likely that the underlying reason for both motility and viability loss was the damage to the cell membrane and the subsequent dissipation of PMF by ROS produced during photocatalysis. Specifically, it is believed that the ROS dissipate the PMF by disrupting the phospholipid bilayer structure of the cell membrane. Thus, motility assays offer a powerful way to discern the effects of photocatalysis in situ and at a single-cell level, which is unlikely to be obtained in most conventional characterization techniques including SEM. Significantly, the assays presented in this work enable the monitoring of temporal changes in the swimming speeds of single cells, which can reveal the rate of dissipation of the PMF and any restorative cellular adaptations as the speeds are proportional to the PMF.

Experiments involving immobilized photocatalysts discussed here clearly indicated that most *E. coli* cells move around the nanowire photocatalysts, without staying adhered to them (Figure 2). Very few instances, if any, of nanowires getting delaminated and attaching to *E. coli* cells were observed (see Videos S1 and S5 in the Supporting Information provided). In fact, in some instances, *E. coli* cells changed their direction of translation following collisions with the nanowires (see also, Videos S1, S2, S5, and S6 in the Supporting Information). This observation is further supported by fluorescence microscopy studies, which indicated that the *E. coli* cells move past nanowires after colliding with them (Video S10). These observations are in sharp contrast with photocatalysis studies involving the use of nanoparticles as photocatalysts. Aeroxide P25 photocatalysts used in the work of Gogniat et al. simply adsorb onto the cell surface.^23^ This observation is significant because the *E. coliK12* strain used by Gogniat and co-workers is related to the one employed in this work (AW405 is derived from K12). In all, the results provide direct evidence for the fact that “constant and sustained contact” or adsorption of the catalyst on the bacterial surface is not a necessary condition for cell inactivation, and that cell inactivation is possible by their repeated collisions with the photocatalyst.^24^ These results also provide direct evidence indicating that photocatalyst shapes and sizes, in addition to their specific surface areas, primarily dictate the quantitative models for water disinfection kinetics. It is therefore not possible to achieve the same kinetics of disinfection when performing photocatalysis using two different photocatalyst morphologies, nanowires and nanoparticles, of similar specific surface areas and under similar experimental conditions.

Based on the above conclusions, the trend of the kinetics of *E. coli* inactivation shown in Figure 4 can thus be explained from a phenomenological perspective. As discussed above, the interaction of the microorganisms and the activated photocatalysts occurs primarily in a collision mode. However, not all collisions occur close to a light-activated site on the catalyst surface. Therefore, a fraction of these collisions would lead to a transfer of ROS from the catalyst to the bacterial cell, leading to partial damage to the cell membranes. Once the damage reaches a threshold (after multiple collisions), the cells lose motility and viability at the same time. In such a case, the rate of inactivation, and thus that of motility loss will be directly dependent on the collision frequency of nanowires and bacteria and the effectiveness of photocatalyst activation by the light source. For a given bacterial cell, this collision frequency will be directly proportional to the number of nanowires present in its vicinity, i.e., the concentration of the nanowires. For the entire control volume, then, this frequency will be proportional to the number of bacterial cells. Therefore, the collision frequency and hence the rate of loss of viability and motility of *E. coli* is proportional to the concentration of motile *E. coli* and the concentration of nanowires activated by UV light. Thus, the collision frequency can be described by
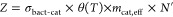
3where σ_bact-cat_ is the average collision cross section, θ(*T*) is a term to capture temperature dependence of collision frequency, *m*_cat,eff_ is the population density of activated catalyst nanowires, and *N*^′^ is the population density of active bacteria.

Assuming a constant fraction (*F*) of nanowire–bacteria collisions lead to a loss in activity, the inactivation rate of cells simplifies to
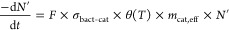
4

In the Petri-dish setup employed in this work, the nanowires are immobilized and are used in a constant concentration, and the light intensity is kept constant. Under these circumstances, the concentration of photocatalyst particles activated by the light and the rate of production of ROS from the photocatalyst remain constant. Thus, the only significant variable affecting the rate of cell inactivation is the cell concentration. Thus, eq 4 simplifies to
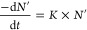
5

This phenomenological model clearly explains the exponential nature of the viable/motile cell concentration vs time curve at the beginning of the treatment (i.e., *t* < 20 min), as described in Sections 3.4 and 3.5. The enhancement in the cell inactivation kinetics observed in the larger scale experiments (i.e., beaker-scale experiments, Figure 5b), relative to those observed in the T-dish-scale experiments, can also be explained on similar lines using the model. Unlike immobilized nanowires, suspensions of both nanowires and *E. coli* cells employed for photocatalysis in the beaker-scale experiments afford a higher frequency of collisions between bacteria and nanowires leading to an enhancement in the *E. coli* inactivation kinetics. The higher collision frequencies are due to the active mixing of the suspensions via magnetic stirring during photocatalysis.

Beyond a treatment time of 20 min, there is an upward deviation from the exponential rate law observed in inactivation of cells (see Figure 5). Possible explanations for this deviation include experimental sampling limitations, ineffective use of ROS generated by the photocatalyst (i.e., some of the ROS may be used up to oxidize the inactivated cells or cell components), or the presence of persister cells in the suspension. In other words, the experimental methods, i.e., spread-plating and motility measurements through video capture, may not be sensitive enough to quantify motility/viability at low active bacterial loads. The competition from the organic materials released into the medium from inactivated cells for the ROS generated by the photocatalyst may also lower the pseudo-first-order rate constant from eq 5. The possible presence of cells resistant to oxidative stress could also explain a fraction of the deviation in kinetics. Nevertheless, the fact remains that a close correlation exists between the motility loss and viability loss at all cell concentrations in this study, and this supports the reliability of the proposed methodology for studying bacterial viability in real time.

## 5 Conclusions

In summary, the phase and fluorescence microscopy and particle-tracking algorithms were employed for deducing a real-time marker that is indicative of *E. coli* inactivation kinetics during TiO_2_ nanowire-assisted photocatalysis, namely, motility. In addition, the set of methods provided information about the mechanisms underlying both *E. coli* inactivation and the mode of interaction of *E. coli* with TiO_2_ nanowires. In all, the rapid quantification of cell activity during photocatalysis presented in this work should help evaluate various catalyst–light source combinations for designing efficient photocatalytic disinfection reactors. Overall, the conclusions of the set of experimental methods presented are as follows: *E. coli* cell motility loss during photocatalysis serves as a good marker for obtaining real-time inactivation kinetics, the interaction of *E. coli* cells with TiO_2_ nanowires is through collisions, and inactivation of *E. coli* occurs through cell membrane integrity loss. The set of methods presented here offer the following advantages over traditional assays used to study bacterial inactivation kinetics during photocatalysis: (i) provide real-time quantification of photocatalysis inactivation kinetics, (ii) provide information about the mode of interaction between the bacteria and the photocatalysts, and (iii) provide information about the mechanisms underlying disinfection of bacteria, i.e., whether oxidative damage is extracellular or intracellular. As motility is employed as the marker for obtaining the kinetics of viability loss, this method is expected to be extendable to other motile microorganisms (e.g., other bacteria, certain fungi, algae, etc.). However, viruses in their planktonic and unlabeled state are unlikely to be motile and observable using optical microscopy; therefore, virus inactivation cannot be studied using this method.

Moreover, this method is not limited by either the size or the chemical composition of the photocatalyst. Nanowires/nanotubes of many photocatalysts are easily observable by optical microscopy. Therefore, bacterial inactivation kinetics associated with nanowire/nanotube-photocatalysis of many materials can be studied using the methodology presented. Even though individual nanoparticles like Aeroxide P25 TiO_2_ nanoparticles are not visible under optical (phase) microscopy, both nanoparticles and nanoparticle agglomerates can be visualized using fluorescence microscopy.^23,55^ Hence, the proposed set of methods can also be used to study bacterial inactivation kinetics associated with Aeroxide P25 TiO_2_ nanoparticle-assisted photocatalysis. It is also essential to add here that the type of excitation employed for photocatalysis is not limited to UV-A light, as is the case in this article. For example, a solar simulator can be used to simulate the excitation of the photocatalysts by sunlight/visible light and study both inactivation kinetics and cell–photocatalyst interactions under those conditions. The aforementioned discussion also sheds light on a few future directions for this research avenue. The study of the inactivation kinetics and mechanisms underlying the inactivation of algae and other pathogenic bacteria will be of interest to the scientific community. More importantly, understanding how Aeroxide P25 TiO_2_ nanoparticles interact with bacteria during photocatalysis will be important to understand whether nanoparticles remain attached to the inactivated bacteria and whether this impacts both their useful lifetimes and the ability to recover and reuse the nanoparticles.

## Data Availability

Examples and details of particle-tracking algorithms and the data sets mentioned in this manuscript will be available from the corresponding author upon request.

## Supporting Information Available

Summary of the experimental procedure and photograph and schematic diagram of the titanium dioxide-coated-Delta-T dish assembly (PDF)
Video micrograph of motile cells before photocatalytic treatment (AVI)
Video micrograph of motile cells after photocatalytic treatment (AVI)
Video micrograph of motile cells before clear control experiment (AVI)
Video micrograph of motile cells after clear control experiment (AVI)
Video micrograph of motile cells before dark control experiment (AVI)
Video micrograph of motile cells after dark control experiment (AVI)
Video micrograph of motile cells before negative control experiment (AVI)
Video micrograph of motile cells after negative control experiment (AVI)
Video micrograph of fluorescent *E. coli* cells (in green) and titanium dioxide nanowire (in red) (AVI)
Video micrograph of fluorescent-tagged flagella of *E. coli* cells (AVI)

## Supplementary Material

ew3c00180_si_001.pdf

ew3c00180_si_002.avi

ew3c00180_si_003.avi

ew3c00180_si_004.avi

ew3c00180_si_005.avi

ew3c00180_si_006.avi

ew3c00180_si_007.avi

ew3c00180_si_008.avi

ew3c00180_si_009.avi

ew3c00180_si_010.avi

ew3c00180_si_011.avi
