# Supplementary material for: Real-Time Deduction of Mechanisms and Kinetics Underlying Photocatalytic Water Disinfection: Cell Motility and Particle Tracking
Source: ACS ES T Water. 2023 Aug 15;3(9):2938–47. doi: 10.1021/acsestwater.3c00180 (PMC10778399; doi:10.1021/acsestwater.3c00180)
Supplement: Supplementary file 1 — ew3c00180_si_001.pdf [file ew3c00180_si_001.pdf]

Real-time Deduction of Mechanisms and Kinetics Underlying Photocatalytic Water Disinfection: Cell Motility and Particle Tracking

Niraj Ashutosh Vidwans[1], Kathy Y. Rhee[1], Pushkar P. Lele[1], Sreeram Vaddiraju[1,2,*]

[1]Artie McFerrin Department of Chemical Engineering, Texas A&M University; College Station, TX 77843, USA

[2]Department of Materials Science and Engineering, Texas A&M University, College Station, TX 77843, USA

*Corresponding Author: Email: sreeram.vaddiraju@tamu.edu; Phone: 979-862-1615

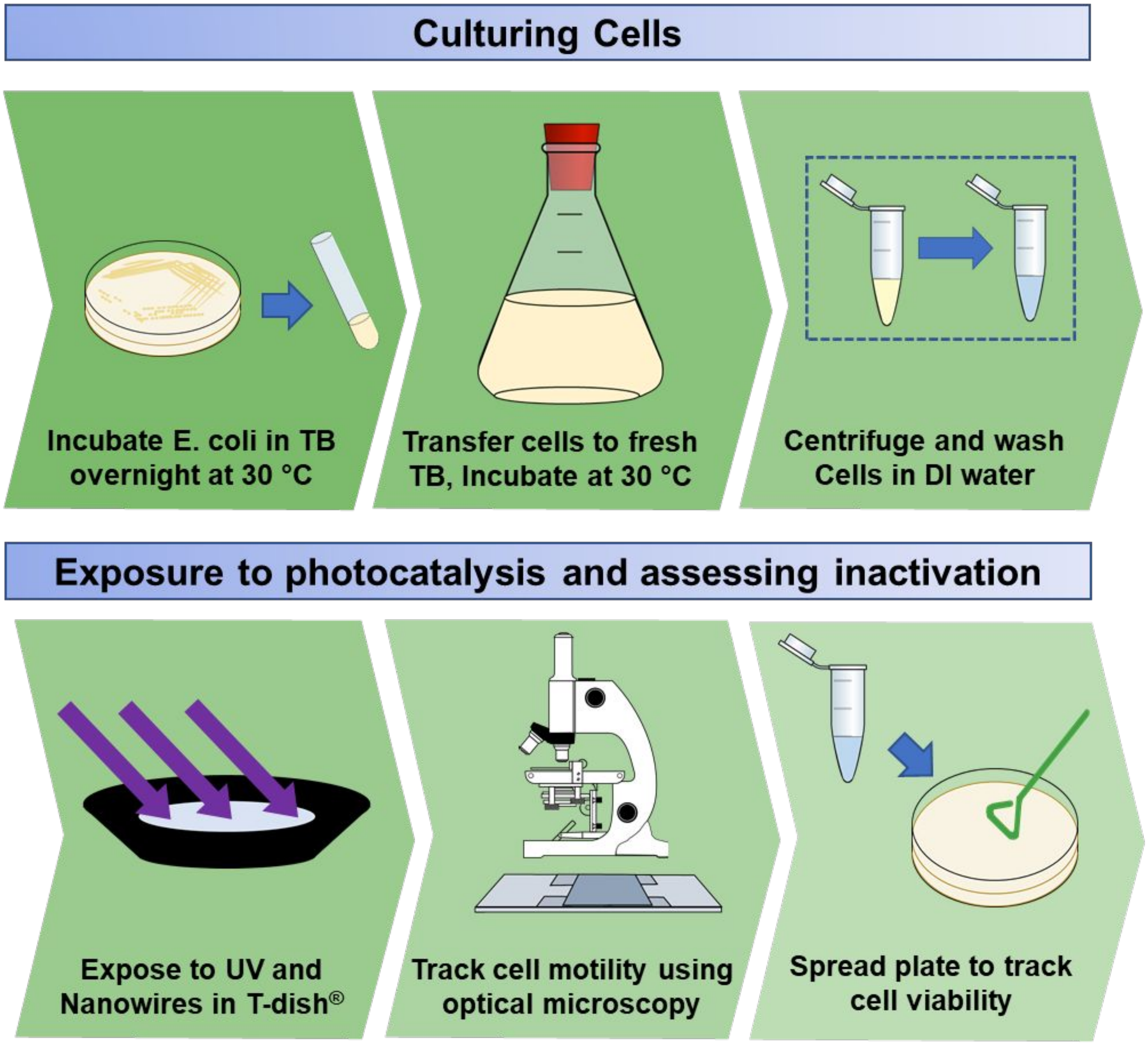

Figure S1. A summary of the experimental procedure followed for simultaneous quantification of cell motility loss and cell viability loss.

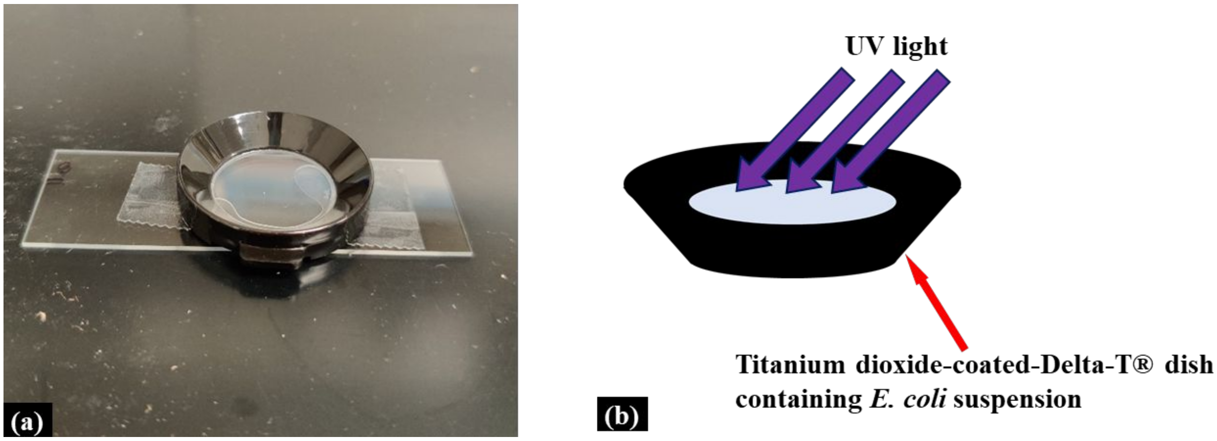

Figure S2. (a) A photograph of the titanium dioxide-coated-Delta-T® dish assembly used in the experiments described in this work (b) a schematic showing the implementation of the photocatalytic reaction.
